# Antigen Mimicry between Infectious Agents and Self or Environmental Antigens May Lead to Long-Term Regulation of Inflammation

**DOI:** 10.3389/fimmu.2013.00314

**Published:** 2013-10-08

**Authors:** Lain Pontes-de-Carvalho, Jose Mengel, Camila A. Figueiredo, Social Changes, Asthma and Allergy in Latin America (SCAALA)’s Study Group, Neuza M. Alcântara-Neves

**Affiliations:** ^1^Centro de Pesquisas Gonçalo Moniz, Fundação Oswaldo Cruz, Salvador, Brazil; ^2^Instituto Oswaldo Cruz, Fundação Oswaldo Cruz, Rio de Janeiro, Brazil; ^3^Instituto de Ciências da Saúde, Universidade Federal da Bahia, Salvador, Brazil; ^4^Instituto de Saúde Coletiva, Universidade Federal da Bahia, Salvador, Brazil; ^5^Faculdade de Medicina, Universidade Federal da Bahia, Salvador, Brazil; ^6^Colegio de Ciencias de La Salud, Universidad San Francisco de Quito, Quito, Ecuador; ^7^Molecular and Biochemical Parasitology, Liverpool School of Tropical Medicine, Liverpool, UK; ^8^London School of Hygiene and Tropical Medicine, London, UK

**Keywords:** autoimmunity, autoreactivity, infection, crossreactivity, IL-10, transforming growth factor-beta, allergy, hygiene hypothesis

## The Past Influences the Present: Infections Induce an Immunological State that Controls the Development of Allergy, Atopy, and Autoimmune Diseases

To account for the increasing prevalences of allergic and autoimmune diseases in populations that partake of good hygiene conditions, and, therefore, are less exposed to pathogens, the hygiene hypothesis proposes that infections favor the long-lasting control of types I and IV hypersensitivity reactions (1). This effect has been ascribed to interleukin (IL-)10 production (2–4). However, type I hypersensitivity can still be kept in check well after the infection has subsided (5), and past infections may protect against the development of autoimmunity (4, 6).

In one study, 613 individuals from two African villages with different prevalences of schistosomiasis caused by *Schistosoma haematobium* were investigated for the presence of circulating anti-nuclear autoantibodies (ANA). ANA levels were lower in the most heavily infected individuals in the low schistosomiasis-prevalence village, although no statistically significant differences among differently infected groups was reported. A statistically significant, but small mean difference of about 3 IU between treated and untreated individuals was observed, a fact that allows one to conclude that ongoing *S. haematobium* infections inhibit the production of ANA. However, at least two pieces of evidence in that study convincingly argue in favor of a stronger effect of past infections in determining ANA levels. Firstly, uninfected individuals’ ANA levels in the high infection-prevalence village were similar to heavily infected individuals’ ANA levels in the low infection-prevalence area. Past infections of uninfected individuals, in the high prevalence area, could account for this finding. Secondly, much lower intensities of infection were observed in more than 22-year-old individuals (mean of approximately 2.5 eggs per 10 mL of urine) than in younger individuals (means ranging from 17.0 to 75.0 eggs per 10 mL of urine) (4). It is very likely, therefore, that the prevalence of infection is also much lower in older individuals, who have been, nevertheless, exposed during a longer time interval than the younger individuals to the parasite. Despite this, no difference in ANA levels among the different age groups were observed, suggesting that past infections down-regulated ANA formation in the older group.

The findings mentioned above provide indirect evidence for the persistence of an expanded population of regulatory cells in the absence of stimulation of the immune system by pathogen-derived antigens. The strength of the immune regulation, however, may increase with the continuous presence of the pathogen or of its antigens. For instance, the prevention of diabetes in NOD mice by the injection of *Trypanosoma cruzi* extract depends on repeated extract injections (Mengel J. et al., unpublished data). It is also reasonable to assume that different diseases may require distinct levels of immune regulation in order to be controlled.

## Do Autoreactive and Allergen-Reactive Immune Regulatory Cells Play a Role in Maintaining an Infection-Triggered Immune Modulatory State?

It is proposed herein a mechanism by which past infections would result in a persistent downregulation of immune-mediated inflammatory reactions. This mechanism would entail the stimulation of autoreactive FoxP3^+^ or FoxP3^−^ regulatory T (Treg) cells or IL-10 – producing B (B10) cells, or bystander stimulation of these cells (7, 8), during immune responses against complex microorganisms. Some of the thousands of foreign epitopes would crossreact with self and lead to the expansion of autoreactive regulatory cells (9). Regulatory cells have indeed been shown to expand during infections (10–13), although it has not been described whether they crossreact with autoantigens. The crossreactive regulatory cells would then be constantly activated by autoantigens, even after the infectious agent had been eliminated. This view agrees with reported evidence that the establishment of peripheral tolerance requires the continuous presence of antigen (14), which, of course, is the case with non-sequestered autoantigens.

**Figure 1:**
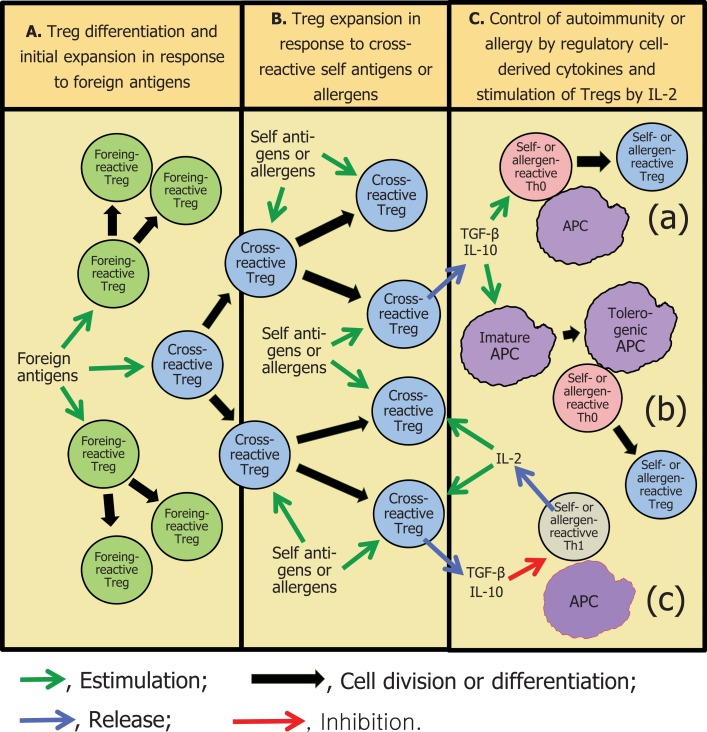
**A possible mechanism for the maintenance of an infection-triggered immune regulatory environment**. **(A)** Foreign, pathogen-derived antigens would activate and initially expand regulatory T cells (Treg) that crossreact with self-antigens and allergens, as well as pathogen-specific regulatory cells. **(B)** Self-antigens or common allergens would further expand and activate the crossreactive Treg cells. **(C)** Regulatory cell-derived IL-10 and TGF-β would inhibit potentially pathogenic immune responses by: (a) diverting the differentiation of naïve, Th0 lymphocytes to T regulatory lineages rather than to other, proinflammatory types (22, 23); (b) promoting the differentiation of tolerogenic antigen-presenting cells (APC) (these cells would lead to the differentiation of regulatory T and B lymphocytes) (24); (c) directly inhibiting the proliferation of and synthesis of proinflammatory cytokines by effector T cells (20, 21). Self- or allergen-reactive Th1 cells would sporadically release the IL-2 necessary to maintain the Treg response, before being controlled by regulatory cytokines. For the sake of clarity, the inflammatory reaction due to infection in **(A)**, the APC required for Treg antigen recognition in **(A,B)**, and regulatory B cells are not depicted in the Figure.

What has been proposed above for autoreactive lymphocytes could well also take place with lymphocytes that recognize ubiquitous foreign antigens. For instance, unless very strict control measures are adopted, most individuals are recurrently exposed to aeroallergens through the respiratory mucosa, in many cases in almost the same degree that they are exposed to autoantigens (15). Moreover, anti-house dust mite antibodies have been shown to crossreact with *Ascaris lumbricoides* aqueous extracts (16). One could, therefore, also propose that mite allergens would maintain mite allergen-reactive regulatory cells, initially activated by crossreactive *A. lumbricoides* antigens, in an active state (Figure 1). Alternatively, pathogen-derived antigens could induce the activation of bystander allergen-specific regulatory cells.

The regulatory cells would act mainly by means of secreted IL-10 and TGF-beta. These cytokines, which have been associated with the control of both autoimmunity (4, 17, 18) and allergic diseases (19), can act by directly inhibiting cytokine synthesis and proliferation of effector lymphocytes (20, 21), by deviating the differentiation of naïve T cells to inducible CD25^+^ regulatory T (iTreg) cells or to Tr1 cells (22, 23), or by conferring a tolerogenic profile to dendritic cells (tolDCs; 19). The tolDCs, in their turn, would induce the development of Tr1 and B10 cells, further intensifying the immune regulatory character of the environment (24) (Figure 1). Another interesting possibility would concern an IL-10- and TGF-beta-induced switching on of IgA synthesis in B cells (25), diverting potentially autoreactive B cells from synthesizing the more pathogenic IgE and IgG isotypes. In accordance with this hypothesis, there is evidence that the majority of human splenic B cells are first stimulated by antigens in gut-associated lymphoid tissues (26), which are skewed toward the presence of regulatory cytokines (27).

The hypothesis proposed above would be consistent both with antigen-specific and non-specific immune regulation. In the first case, the regulatory lymphocytes would act specifically on autoantigen- and allergen-reactive T cells (in the case of Treg cells by binding to the same antigen-presenting cells that memory effector lymphocytes or naïve lymphocytes are bound to). Non-specific immune regulation would perhaps result from increased extracellular concentrations of regulatory cytokines (23). As far as the authors’ knowledge goes, no evidence for antigen-specificity or non-specificity of the phenomena dealt with in the hygiene hypothesis has been provided in the literature.

A particular type of regulatory cell that could be involved in an infection-triggered immune regulatory chain of events is the IL-10-secreting Th1 cell (14, 28, 29), since, contrasting with other regulatory cells, it is not anergic (28), and would, therefore, be more easily driven to expansion. IL-10-secreting Th1 cells have been shown to expand during infections, have been associated with the control of allergy and autoimmunity (14, 28) and are believed to result from cytokine synthesis switching in effector T cells (29). They would, therefore, unlike other Tregs, be formed in proinflammatory conditions, something that would entail their presence in large numbers in animals with infectious diseases. In fact, high proportion of the cells that infiltrate muscle tissue in *T. cruzi*-infected mice synthesizes both IL-10 and interferon (IFN-)gamma (Mengel J. et al., unpublished data).

Supporting the hypothesis proposed herein, it has been observed that peripheral blood mononuclear cells from individuals who have been infected by helminths produce IL-10 in the absence of *in vitro* stimulation, whereas they produced no additional IL-10 when stimulated *in vitro* by helminth antigens (30). This finding could be explained by the IL-10 being produced by crossreactive regulatory T or B cells that would already had been stimulated by autoantigens *in vivo*. Crossreactivities between helminth antigens and autoantigens have indeed been described (31–35).

It is proposed in this Opinion that the IL-2 required for the maintenance of an immune regulatory state would derive from Th1 cells. These cells would be activated by autoantigens or allergens when the immune regulatory cells had faltered due to the very lack of IL-2. One could therefore envisage, therefore, that a functional immune regulatory state would be maintained by the recurrent but transient activation of potentially pathogenic autoreactive or allergen-reactive effector Th1 lymphocytes. These lymphocytes would then be immediately controlled, before they could cause overt disease, by the prompt activation of previously expanded immune regulatory cells by the released IL-2. An alternance of short periods of potentially pathogenic immune responses and long periods in which these responses were kept in check would therefore ensue.

Some autoimmune diseases, such as collagen diseases and multiple sclerosis, have periods of clinical activity separated by remission intervals. At least in some occasions this could reflect disturbances in the immunological environment, whose regulatory potential would augment following periods of disease activity in which IL-2 would be made available.

## The Parasites’ Good Luck: Some Crossreactivity May Protect Them from the Immune System, No Crossreactivity May Not be an Option at all

An initial requisite for an infection-triggered regulatory state be maintained in the absence of infection, as proposed in this Opinion, is that the infecting organism should crossreact with self or with a foreign antigen that the host would be in almost permanent contact with. This requisite would perhaps favor the occurrence of the phenomenon in infections by complex pathogens, of which helminths, of course, are the best representatives, since the number of different epitopes present in an organism would be a function of the number of different proteins synthesized by it, which in its turn tends to increase with the organism complexity. However, there is not, *a priori*, *any* reason why the phenomenon would not occur with infections by non-helminthic pathogens.

The number of different epitopes present in complex organisms, constituted by specialized cells, such as helminths, mites, or human beings, could indeed be enormous. Even in a simpler unicellular microorganism, such as *Trypanosoma brucei*, a total of 8,960 mRNA transcripts have been predicted (36), and, therefore, there is a potential for the same number of different antigens to be expressed. Since at least one to four epitopes per polypeptide have been shown to be presented to CD4^+^ lymphocytes by antigen-presenting cells in a single individual (37–39), the probability that at least a few among the thousands of a complex organism epitopes would be similar to a few of the thousands of epitopes of another complex organism could be very high indeed. This probability of occurrence of crossreaction is greatly increased in function of the degenerate recognition of peptides by the CD4^+^ T-cell antigen receptor (TCR), i.e., complete identity between peptides is not a requisite for crossreactivity (39), and, last but not least, to sequence homologies among phylogenetically related proteins.

Several examples of crossreactivity of pathogens with autoantigens have been reported [e.g., (31–35, 39–45)], some of which due to phylogenetic homologies (32–34, 42, 44). A particular study has shown that, depending on the HLA allele, 80–290 MHC II-binding peptides from *Mycobacterium tuberculosis* crossreact with human autoantigens (45).

On the other hand, it is conceivable that a pathogen would face a compromised immune response even when only one of its antigens stimulated pre-expanded autoreactive immune regulatory cells, through the mechanism of linked or bystander suppression (7, 8). Thus, there could be, in fact, a selective pressure for pathogens to expose epitopes crossreacting with self to the host immune system.

Another requisite for the intensification of the immune regulatory state by infections, namely the infectious agents inducing the production of regulatory cytokines, could also favor a major role played by helminth infections, as these are often associated with the so-called “modified Th2-cell responses,” in which IL-10 is produced concomitantly with IL-4 and IL-13 (3).

## Testable Predictions of the Proposed Hypothesis

If the immune regulatory cells are maintained in an active state or as memory cells by autoantigens and customary allergens, they should be specific for these antigens. This is amenable to be experimentally tested, as animals that had had past chronic infections should respond normally to the immunization with unrelated foreign antigens but be less prone to the experimental induction of autoimmune diseases. Another question to be answered is whether the regulatory cells, in addition to react with self and allergenic antigens, would also react with crossreactive infectious agent antigens. A possible approach to answer it could involve the use of flow cytometry to enumerate Treg cells capable of binding to MHC tetramers associated with crossreactive peptides, or to enumerate crossreactive antigen-binding B10 cells.

## References

[1] Wills-Karp M Santeliz J Karp CL The germless theory of allergic disease: revisiting the hygiene hypothesis. Nat Rev Immunol (2001) 1:69–75 10.1038/35095579 11905816

[2] Yazdanbakhsh M Kremsner PG van Ree R Allergy, parasites, and the hygiene hypothesis. Science (2002) 296:490–4 10.1126/science.296.5567.490 11964470

[3] Maizels RM Yazdanbakhsh M Immune regulation by helminth parasites: cellular and molecular mechanisms. Nat Rev Immunol (2003) 3:733–44 10.1038/nri1183 12949497

[4] Mutapi F Imai N Nausch N Bourke CD Rujeni N Mitchell KM *Schistosome* infection intensity is inversely related to auto-reactive antibody levels. PLoS ONE (2011) 6:e19149 10.1371/journal.pone.0019149 21573157 PMC3089602

[5] Rodrigues LC Newcombe PJ Cunha SS Alcantara-Neves NM Genser B Cruz AA Early infection with *Trichuris trichiura* and allergen skin test reactivity in later childhood. Clin Exp Allergy (2008) 38:1769–77 10.1111/j.1365-2222.2008.03027.x 18547322

[6] Plot L Amital H Barzilai O Ram M Nicola B Shoenfeld Y Infections may have a protective role in the etiopathogenesis of celiac disease. Ann N Y Acad Sci (2009) 1173:670–4 10.1111/j.1749-6632.2009.04814.x 19758214

[7] Honey K Cobbold SP Waldmann H Dominant tolerance and linked suppression induced by therapeutic antibodies do not depend on Fas-FasL interactions. Transplantation (2000) 69:1683–9 10.1097/00007890-200004270-00026 10836381

[8] Apostolou I Verginis P Kretschmer K Polansky J Hühn J von Boehmer H Peripherally induced Treg: mode, stability, and role in specific tolerance. J Clin Immunol (2008) 28:619–24 10.1007/s10875-008-9254-8 18841451

[9] Bluestone JA Abbas AK Natural versus adaptive regulatory T cells. Nat Rev Immunol (2003) 3:253–7 10.1038/nri1032 12658273

[10] Kared H Fabre T Bédard N Bruneau J Shoukry NH Galectin-9 and IL-21 mediate cross-regulation between Th17 and Treg cells during acute hepatitis C. PLoS Pathog (2013) 9:e1003422 10.1371/journal.ppat.1003422 23818845 PMC3688567

[11] McBride A Konowich J Salgame P Recruitment of Foxp3+ T regulatory cells to the lungs in chronic *Mycobacterium tuberculosis* infection requires Toll-like receptor 2. PLoS Pathog (2013) 9:e1003397 10.1371/journal.ppat.1003397 23785280 PMC3681744

[12] Chevalier MF Weiss L The split personality of regulatory T cells in HIV infection. Blood (2013) 121:29–37 10.1182/blood-2012-07-409755 23043072

[13] Shan Y Liu J Pan Y-Y Jiang Y-J Shang H Cao Y-M Age-related CD4 (+) CD25(+) Foxp3(+) regulatory T-cell responses during *Plasmodium berghei* ANKA infection in mice susceptible or resistant to cerebral malaria. Korean J Parasitol (2013) 51:289–95 10.3347/kjp.2013.51.3.289 23864739 PMC3712102

[14] Meiler F Zumkehr J Klunker S Rückert B Akdis CA Akdis M In vivo switch to IL-10-secreting T regulatory cells in high dose allergen exposure. J Exp Med (2008) 205:2887–98 10.1084/jem.20080193 19001136 PMC2585856

[15] Baqueiro T Carvalho FM Rios CF dos Santos NM Alcântara-Neves NM Medical Student Group Dust mite species and allergen concentrations in beds of individuals belonging to different urban socioeconomic groups in Brazil. J Asthma (2006) 43:101–5 10.1080/02770900500497958 16517425

[16] Ponte JC Junqueira SB Veiga RV Barreto ML Pontes-de-Carvalho LC Alcântara-Neves NM A study on the immunological basis of the dissociation between type I-hypersensitivity skin reactions to *Blomia tropicalis* antigens and serum anti-*B. tropicalis* IgE antibodies. BMC Immunol (2011) 12:34 10.1186/1471-2172-12-34 21631925 PMC3118201

[17] Di Marco R Xiang M Zaccone P Leonardi C Franco S Meroni P Concanavalin A-induced hepatitis in mice is prevented by interleukin (IL)-10 and exacerbated by endogenous IL-10 deficiency. Autoimmunity (1999) 31:75–83 10.3109/08916939908994050 10680745

[18] Burkhart C Liu GY Anderton SM Metzler B Wraith DC Peptide-induced T cell regulation of experimental autoimmune encephalomyelitis: a role for IL-10. Int Immunol (1999) 11:1625–34 10.1093/intimm/11.10.1625 10508180

[19] Nabe T Ikedo A Hosokawa F Kishima M Fujii M Mizutani N Regulatory role of antigen-induced interleukin-10, produced by CD4 (+) T cells, in airway neutrophilia in a murine model for asthma. Eur J Pharmacol (2012) 677:154–62 10.1016/j.ejphar.2011.12.020 22209878

[20] Taga K Tosato G IL-10 inhibits human T cell proliferation and IL-2 production. J Immunol (1992) 148:1143–8 1737931

[21] Gorelik L Flavell RA Abrogation of TGFbeta signaling in T cells leads to spontaneous T cell differentiation and autoimmune disease. Immunity (2000) 12:171–81 10.1016/S1074-7613(00)80170-3 10714683

[22] Gorelik L Constant S Flavell RA Mechanism of transforming growth factor beta-induced inhibition of T helper type 1 differentiation. J Exp Med (2002) 195:1499–505 10.1084/jem.20012076 12045248 PMC2193549

[23] Groux H Type 1 T-regulatory cells: their role in the control of immune responses. Transplantation (2003) 75:8S–12S 10.1097/01.TP.0000067944.90241.BD 12819483

[24] Volchenkov R Karlsen M Jonsson R Appel S Type 1 regulatory T cells and regulatory B cells induced by tolerogenic dendritic cells. Scand J Immunol (2013) 77:246–54 10.1111/sji.12039 23442246

[25] Fayette J Dubois B Vandenabeele S Bridon JM Vanbervliet B Durand I Human dendritic cells skew isotype switching of CD40-activated naive B cells towards IgA1 and IgA2. J Exp Med (1997) 185:1909–18 10.1084/jem.185.11.1909 9166420 PMC2196343

[26] Vossenkämper A Blair PA Safinia N Fraser LD Das L Sanders TJ A role for gut-associated lymphoid tissue in shaping the human B cell repertoire. J Exp Med (2013) 210:1665–74 10.1084/jem.20122465 23940259 PMC3754866

[27] Izcue A Coombes JL Powrie F Regulatory lymphocytes and intestinal inflammation. Annu Rev Immunol (2009) 27:313–38 10.1146/annurev.immunol.021908.132657 19302043

[28] Cardone J Le Friec G Vantourout P Roberts A Fuchs A Jackson I Complement regulator CD46 temporally regulates cytokine production by conventional and unconventional T cells. Nat Immunol (2010) 11:862–71 10.1038/ni.1917 20694009 PMC4011020

[29] Cope A Le Friec G Cardone J Kemper C The Th1 life cycle: molecular control of IFN-γ to IL-10 switching. Trends Immunol (2011) 32:278–86 10.1016/j.it.2011.03.010 21531623

[30] Figueiredo CA Barreto ML Rodrigues LC Cooper PJ Silva NB Amorim LD Chronic intestinal helminth infections are associated with immune hyporesponsiveness and induction of a regulatory network. Infect Immun (2010) 78:3160–7 10.1128/IAI.01228-09 20404082 PMC2897394

[31] Braun G McKechnie NM Gürr W Molecular and immunological characterization of hr44, a human ocular component immunologically cross-reactive with antigen Ov39 of *Onchocerca volvulus*. J Exp Med (1995) 182:1121–31 10.1084/jem.182.4.1121 7561685 PMC2192280

[32] Colebrook AL Lightowlers MW Serological reactivity to heat shock protein 70 in patients with hydatid disease. Parasite Immunol (1997) 19:41–6 10.1046/j.1365-3024.1997.d01-141.x 9121839

[33] Gounni AS Spanel-Borowski K Palacios M Heusser C Moncada S Lobos E Pulmonary inflammation induced by a recombinant gamma-glutamyl transpeptidase homolog: involvement of humoral autoimmune responses. Mol Med (2001) 7:344–54 11474580 PMC1950044

[34] Carvalho-Queiroz C Cook R Wang CC Correa-Oliveira R Bailey NA Egilmez NK Cross-reactivity of *Schistosoma mansoni* cytosolic superoxide dismutase, a protective vaccine candidate, with host superoxide dismutase and identification of parasite-specific B epitopes. Infect Immun (2004) 72:2635–47 10.1128/IAI.72.5.2635-2647.2004 15102772 PMC387882

[35] Radovic I Gruden-Movsesijan A Ilic N Mostarica-Stojkovic M Sofronic-Milosavljevic L *Trichinella spiralis* shares epitopes with human autoantigens. Mem Inst Oswaldo Cruz (2012) 107:503–9 10.1590/S0074-02762012000400010 22666861

[36] Kolev NG Franklin JB Carmi S Shi H Michaeli S Tschudi C The transcriptome of the human pathogen *Trypanosoma brucei* at single-nucleotide resolution. PLoS Pathog (2010) 6:e1001090. 10.1371/journal.ppat.1001090 20838601 PMC2936537

[37] Cole GA Tao T Hogg TL Ryan KW Woodland DL Binding motifs predict major histocompatibility complex class II-restricted epitopes in the Sendai virus M protein. J Virol (1995) 69:8057–60 7494321 10.1128/jvi.69.12.8057-8060.1995 PMC189753

[38] Novak EJ Liu AW Gebe JA Falk BA Nepom GT Koelle DM Tetramer-guided epitope mapping: rapid identification and characterization of immunodominant CD4+ T cell epitopes from complex antigens. J Immunol (2001) 166:6665–70 11359821 10.4049/jimmunol.166.11.6665

[39] Iwai LK Juliano MA Juliano L Kalil J Cunha-Neto E T-cell molecular mimicry in Chagas disease: identification and partial structural analysis of multiple cross-reactive epitopes between *Trypanosoma cruzi* B13 and cardiac myosin heavy chain. J Autoimmun (2005) 24:111–7 10.1016/j.jaut.2005.01.006 15829403

[40] Van Voorhis WC Eisen H Fl-160. A surface antigen of *Trypanosoma cruzi* that mimics mammalian nervous tissue. J Exp Med (1989) 169:641–52 10.1084/jem.169.3.641 2466939 PMC2189289

[41] Williams KM Raybourne RB Demonstration of cross-reactivity between bacterial antigens and class I human leukocyte antigens by using monoclonal antibodies to *Shigella flexneri*. Infect Immun (1990) 58:1774–81 2187807 10.1128/iai.58.6.1774-1781.1990 PMC258722

[42] Li SG Quayle AJ Shen Y Kjeldsen-Kragh J Oftung F Gupta RS *Mycobacteria* and human heat shock protein-specific cytotoxic T lymphocytes in rheumatoid synovial inflammation. Arthritis Rheum (1992) 35:270–81 10.1002/art.1780350305 1371388

[43] Ellis NM Li Y Hildebrand W Fischetti VA Cunningham MW T cell mimicry and epitope specificity of cross-reactive T cell clones from rheumatic heart disease. J Immunol (2005) 175:5448–56 16210652 10.4049/jimmunol.175.8.5448

[44] Glaser AG Menz G Kirsch AI Zeller S Crameri R Rhyner C Auto- and cross-reactivity to thioredoxin allergens in allergic bronchopulmonary aspergillosis. Allergy (2008) 63:1617–23 10.1111/j.1398-9995.2008.01777.x 19032234

[45] Chodisetti SB Rai PK Gowthaman U Pahari S Agrewala JN Potential T cell epitopes of *Mycobacterium tuberculosis* that can instigate molecular mimicry against host: implications in autoimmune pathogenesis. BMC Immunol (2012) 13:13 10.1186/1471-2172-13-13 22435930 PMC3359254

